# In‐Vivo Hand Mass Determination and an Anthropometric Investigation on Segment Length and Radius for Prosthetic Segment Design

**DOI:** 10.1049/htl2.70078

**Published:** 2026-04-08

**Authors:** Panagiotis Tsakonas, Neil D. Evans, Joseph Hardwicke, Shervanthi Homer‐Vanniasinkam, Michael J. Chappell

**Affiliations:** ^1^ School of Engineering University of Warwick Coventry UK; ^2^ Institute of Applied & Translational Technologies in Surgery Coventry UK; ^3^ University College London, School of Engineering London UK

**Keywords:** anthropometric measurements, cylindrical approximation, finger amputation, hand mass, upper limb

## Abstract

This paper aims to demonstrate the validity of the use of a cylindrical approximation for human fingers and a parallelepiped approximation of the palm, from a modelling perspective, for the determination of the mass of the hand. The goal is to provide an intuitive way of determining the length and radius of missing segments from partial hand amputees based on palm dimensions and determine the corresponding mass based on the previous two approximations. In‐vivo hand mass measurements were taken from 23 able‐bodied participants using Archimedes’ water displacement method to verify the geometric approximations used. Furthermore, an anthropometric investigation on how segment length and radius change with respect to palm dimensions was undertaken and the estimates found, which can then be used in supporting the design of prosthetic segments in a personalised context.

## Graphical abstract

This paper determines the hand mass using cylindrical and parallelepiped geometrical approximations. Additionally, it investigates how dimensions of fingers change with respect to the palm to facilitate prosthetic digit development for partial hand amputees.

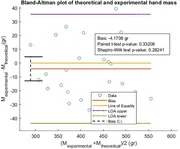

## 1 Introduction

Partial or complete amputation of any limb can prove to be one of the most life‐changing events that can happen to an individual. It has been reported that partial amputation of the hand is about three to six times greater in men than in women and there are approximately 1.9 partial amputations for every 100,000 subjects aged between 25 and 60 years old on an annual basis [1]. For the hand, amputation of even a very small part of a finger can cause physiological and functional loss alongside a reduction of the grip and pinch forces that can be exerted while performing activities of everyday living [2, 3]. To address these challenges, it is crucial to develop a prosthesis that can mimic the functionality and morphology of a natural hand as closely as possible. However, designing hand prostheses is a non‐trivial task. Different types of prostheses are used depending on the level of amputation and this range from cosmetic ones to multi degrees of freedom mechanisms [4]. This range shows how complex designing a universally acceptable prosthesis can be. Not only that but also determining the dimensions of missing segments is another important factor in designing prostheses. There are few papers in the literature that provide anthropometric measurements for human upper limbs that can be used in the design of prostheses for complete or partial amputations. In a PubMed search, using the keywords ‘anthropometric measurements’ AND ‘upper limb’ AND ‘amputation’, just 9 results were achieved for papers published between 1999 and 2022 and none of these relate to amputation. Moreover, in an IEEE Xplore advanced search, inputting the same keywords yielded zero results. This provides a clear indication that there is a gap in the literature for the provision of anthropometric measurements of the upper limb and how these can be translated into the design of prostheses for partial hand amputees. Previous research [5, 6], focus on the biomechanics of the hand/upper limbs. Only two papers have been found in the literature that include discussion on anthropometric measurements, namely, Buchholz et al. [7] and Garrett J. [8]. The former is perhaps the most relevant in terms of providing a scaling equation for such measurements, but then only for segment length, using data from just six cadavers. The latter paper investigated the differences between the upper limb dimensions of male and female air force flight personnel and did not provide any form of scaling equations for anthropometric measurements. This paper aims to provide an intuitive way of determining the length and radius of missing segments from partial hand amputees based on their palm dimensions and using these dimensions to determine the corresponding mass of the missing segment based on a cylindrical approximation.

This paper will attempt to outline the theoretical structure used for determining the mass of the segments and palm of the hand based on cylindrical and parallelepiped approximations, respectively. It will introduce the criteria used for estimating segment length and radius and the dimensions of the palm and describe the experimental design utilised for in vivo hand mass determination. Section three provides the results obtained from the experiments and introduces novel scaling functions for determining segment length and radius from palm dimensions. In section four, a discussion of the results of the study is presented. Section five provides conclusions on the study performed.

## 2 Methods

The human finger segments are approximated as uniform cylinders, and their mass is calculated using the density formula as shown in (1),

(1) $$\;m_{i}=\pi \rho R_{i}^{2}L_{i}$$

where $m_{i},\;\;R_{i}\;\mathrm{and}\;\;L_{i}$ are the mass, the radius and the length of finger segment i respectively, and ρ is the density of the human hand which is selected as $\rho =1.16\;\mathrm{gr}/cm^{3}$ from [9], as the mean density between the left and right hand.

The mass of the palm, $m_{p}$, was calculated using a parallelepiped approximation method, as shown in (2),

(2) $$\;m_{p}=\mathrm{PL}*\mathrm{HB}*\mathrm{HW}*\rho$$

where PL,HBandHW are the length, the breadth and the width of the palm respectively and ρ is the density of the human hand. Using (1) and (2) a theoretical hand mass can be obtained from the summation of the individual masses of the finger segments and palm from their respective dimensions. Using this approach, we introduce criteria to perform repeatable anthropometric measurements.

For the most distal segment of a finger, its length is measured between the most prominent crease on the dorsal side of the distal interphalangeal joint, which is present during flexion, and the tip of the finger.

For the middle finger segment, its length is measured between the most prominent crease on the dorsal side of the proximal interphalangeal joint and the most prominent crease on the dorsal side of the distal interphalangeal joint, which is present during flexion.

For the proximal finger segment, its length is measured between the perceived gap of the metacarpophalangeal joint, which can be felt via palpation, and the most prominent crease on the dorsal side of the proximal interphalangeal joint.

The radius of each finger segment is determined by measuring the length between the ulnar and radial sides of the segment at its midpoint and dividing this by two. To mitigate the impact of soft tissue compressibility on measurement accuracy, the calliper was placed gently against each segment, ensuring minimal disturbance to the soft tissue and allowing for more reliable readings.

**FIGURE 1:**
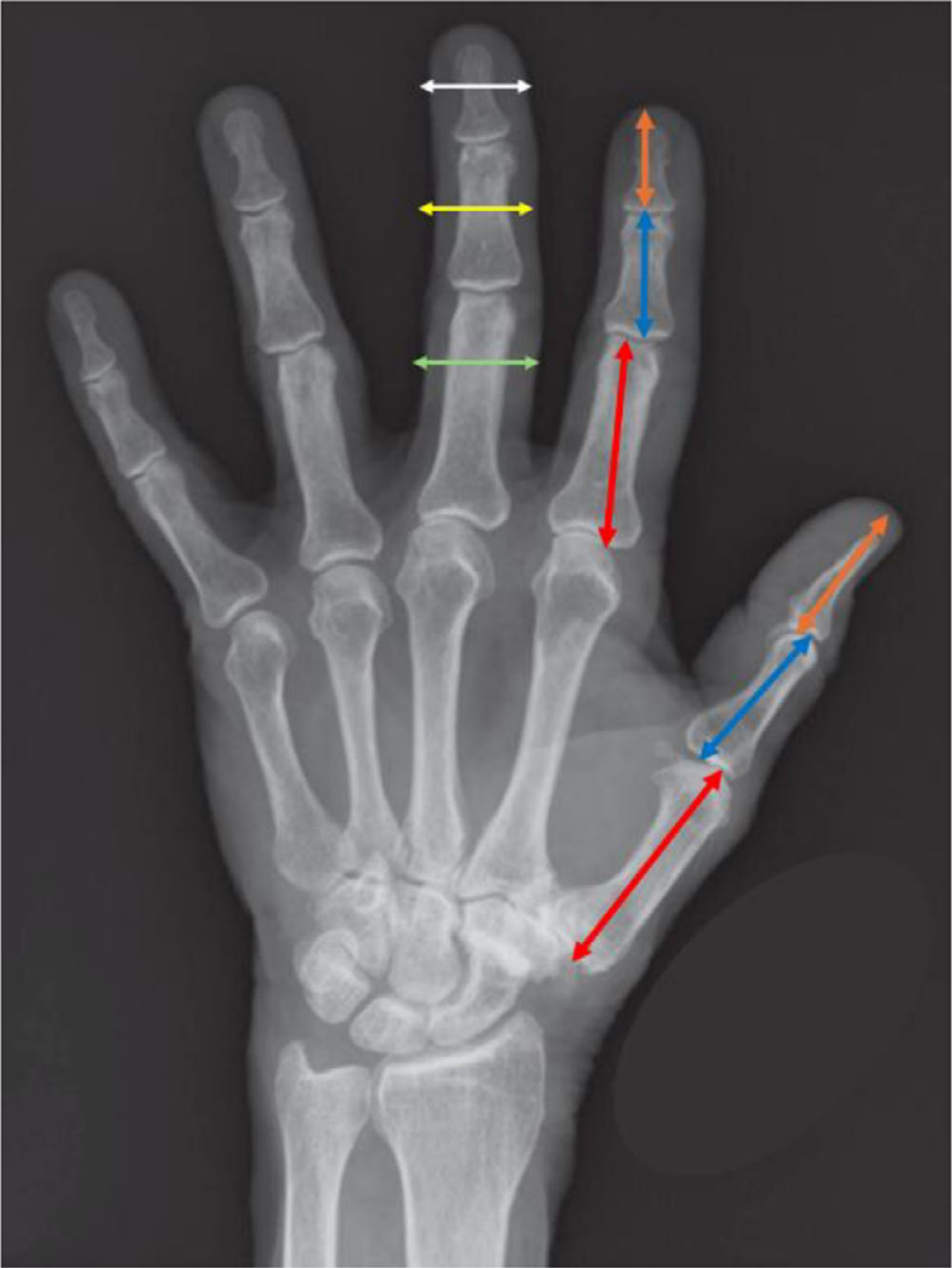
Landmarks for measurements of the finger segments. The orange arrow corresponds to the landmarks for the measurement of the distal segment. The blue arrow corresponds to the landmarks used for the medial segment, the red arrow corresponds to the landmarks used for the measurement of the proximal segment and the white arrow shows the landmarks for the measurement of the diameter of the distal segment. The yellow arrow shows the landmarks for the measurement of the diameter of the middle segment and the green arrow shows the landmarks for the measurement of the diameter of the proximal segment. [Correction added on 6 May 2025, after first online publication: The figure 1 has been updated in this version.]

Figure 1 shows the relevant landmarks for the measurement of the segment length and radius finger segments.

**FIGURE 2:**
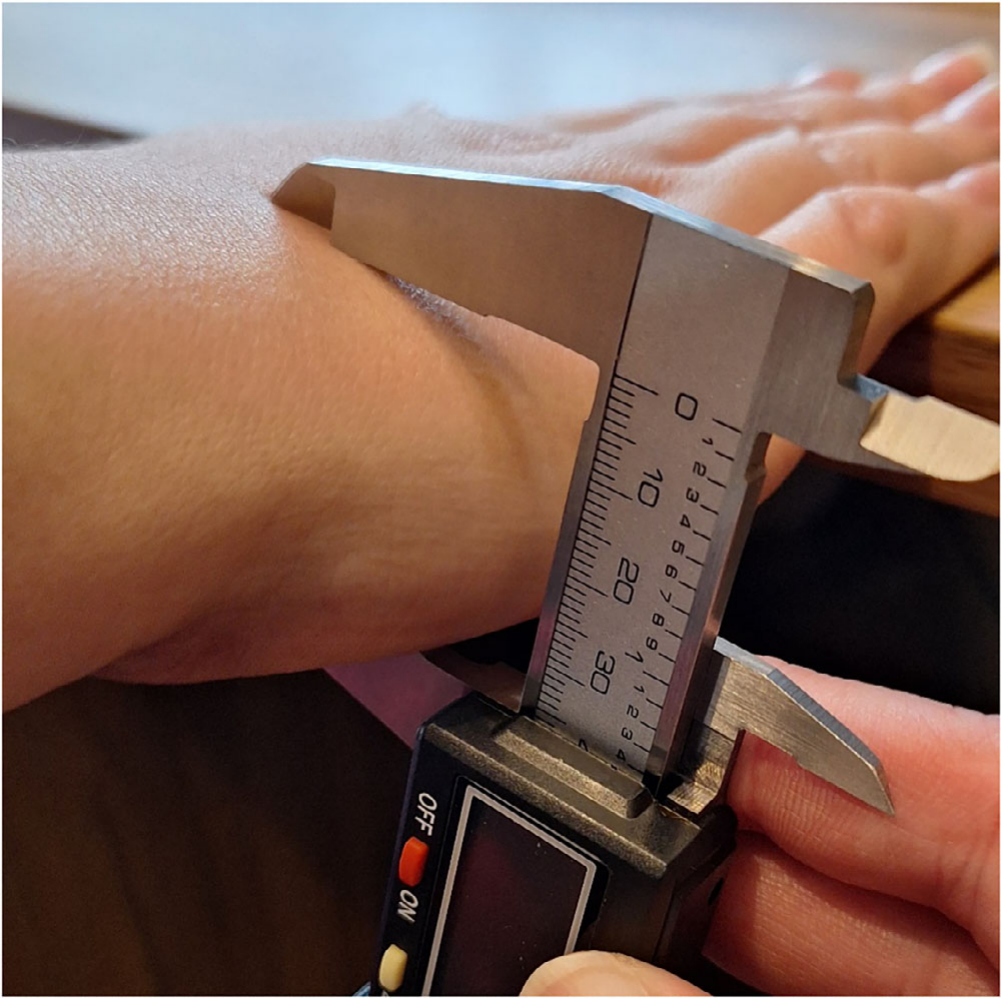
Measurement method for HW. The blue arrows indicate the distance between the dorsal and palmar sides of the palm, marking the measurement location.

**FIGURE 3:**
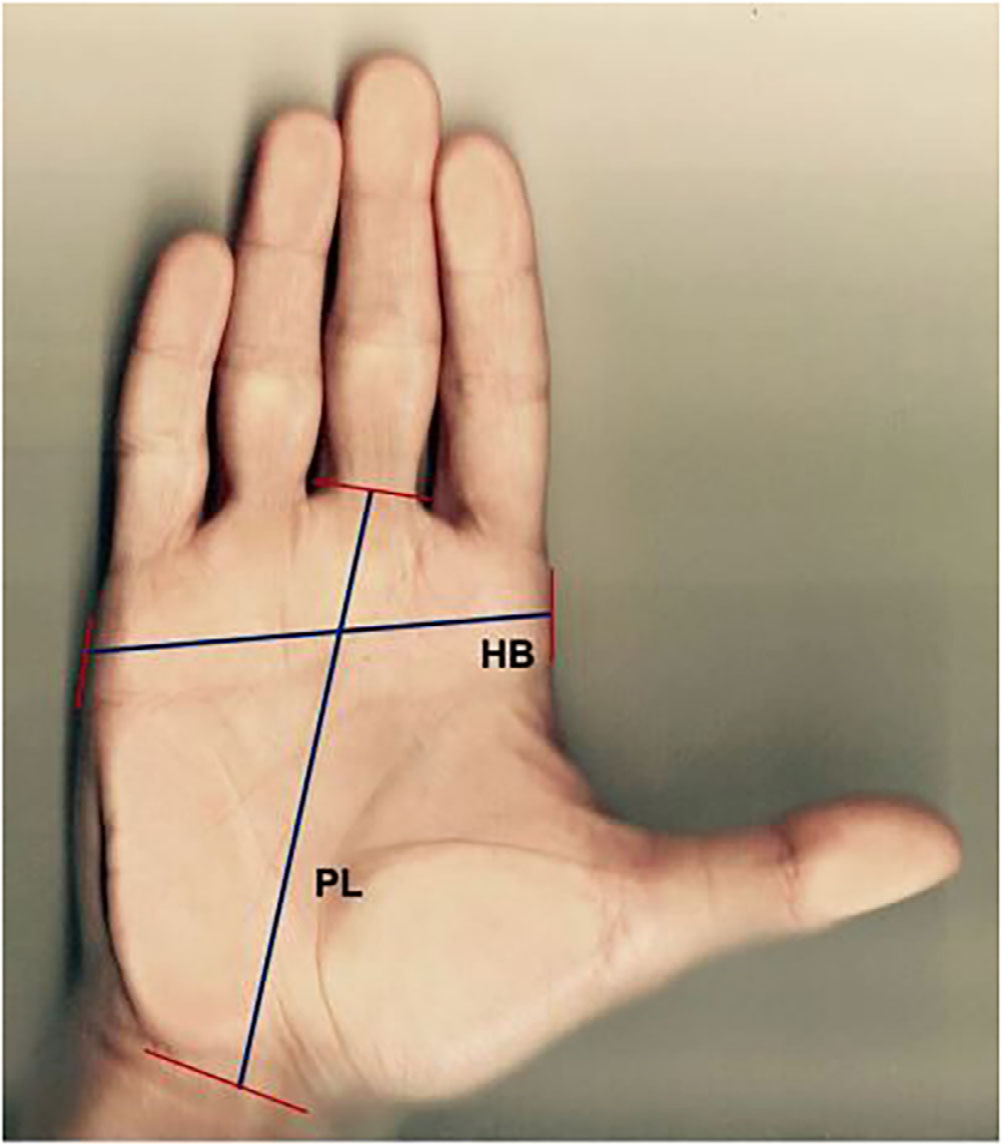
Measurement landmarks for palm length (PL) and hand breadth (HB). Image taken from [10].

The palm length (PL) is measured from the midpoint between the radial and ulnar styloid processes up to the gap for the proximal joint of the middle finger. Hand breadth (HB) is measured between the ulnar side of the proximal joint of the little finger and the radial side of the proximal joint of the index finger, with all fingers in extension. Hand width (HW) is measured from the ulnar side of the metacarpal bone of the little finger as the vertical distance between the palmar and dorsal sides of the palm. To mitigate the impact of soft tissue compressibility on measurement accuracy, the calliper was placed gently between the palmar and dorsal sides of the palm, ensuring minimal compression of the surrounding tissue. Figure 2 shows the placement of the calliper for the HW measurement. Figure 3 shows the landmarks used for the measurements of the PL and HB [10].

**FIGURE 4:**
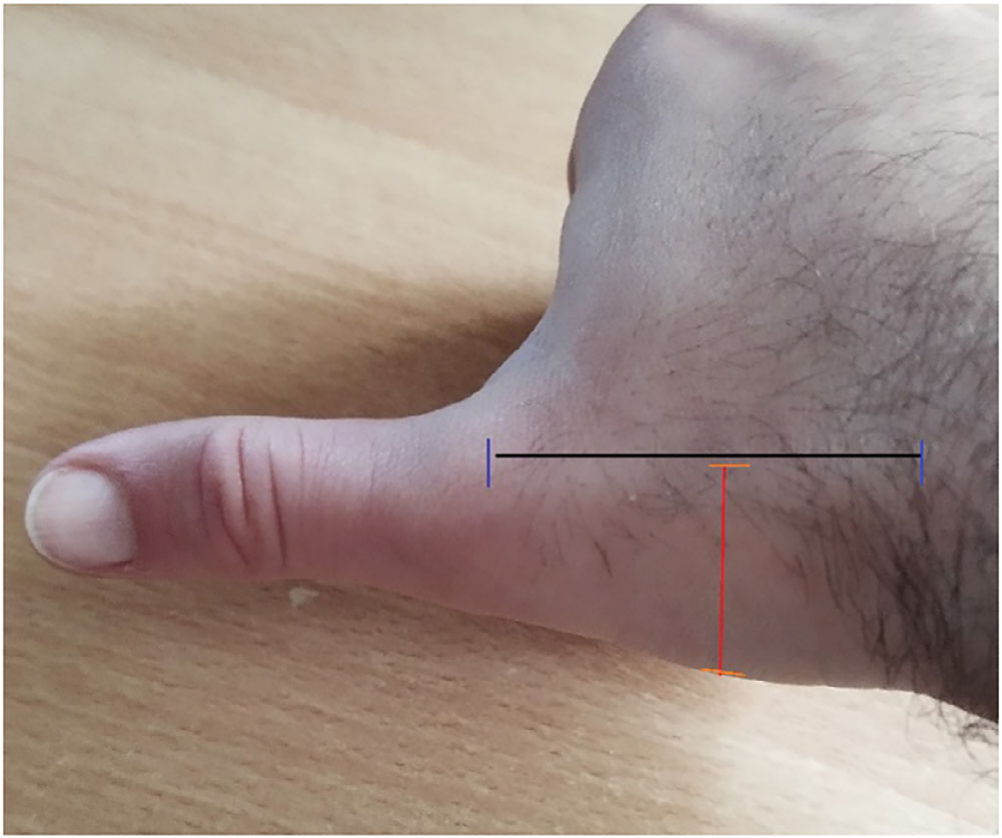
The black line shows the distance between the trapezium bone and the metacarpal segment, and the red line shows the length between the dorsal and palmar sides of the metacarpal segment, which is used to determine the segment radius.

The thumb's metacarpal segment length is measured from the gap between the trapezium bone and the metacarpal segment up to the gap for the thumb's proximal segment, as shown in Figure 4 (the black line). The radius of the thumb metacarpal segment is determined by measuring the length between the dorsal and palmar sides of the metacarpal segment, at its midpoint, and dividing this by two, as shown in Figure 4 (the red line).

To experimentally verify the total hand mass, Archimedes’ principle of water displacement was used. A container with known volume was completely filled with tap water at room temperature. Participants were then instructed to place their hand into the container up to the level of their wrist, which is located between the ulnar and radial styloid processes. Then, the displaced water was simultaneously collected in a different container and was weighed using a digital scale. The mass of the hand is calculated using the density formula as shown in (3):

(3) $$\;m_{\mathrm{hand}}=\frac{\rho }{\rho_{\mathrm{water}\;}}\;m_{\mathrm{displaced}}$$

The density of the water was set to $\rho_{\mathrm{water}}=0.997\;\mathrm{gr}/cm^{3}$ and ρ is the above‐mentioned density of the human hand.

This study was granted full approval by the Biomedical and Scientific Research Ethics Committee (BSREC) at the University of Warwick (reference: BSREC 55/21‐22), on 15/03/2022. Twenty‐three able‐bodied participants were recruited for this study, and relevant measurements of their finger segments and palms were obtained using a digital calliper, following the guidelines outlined in the methods section for identifying key landmarks. This study was designed as a proof of concept, and its sample size was selected to exceed that considered in [7], which included data from 6 cadavers. Additionally, an appropriate sample size calculation was conducted based on the method described in [11] using an expected correlation estimate between the two methods of 0.6. This analysis indicated a recommended sample size of 19 subjects. Consequently, our chosen sample size is deemed to be sufficient to achieve statistical significance and support model robustness. The calliper‐derived measurements were taken from each participant's dominant hand and subsequently used in Equations (1) and (2) to calculate the theoretical hand mass. To experimentally determine the hand mass, each participant placed their hand in the small container five times, and a mean and a standard deviation of the measurements were then performed from these five measurements.

## 3 Results

**FIGURE 5:**
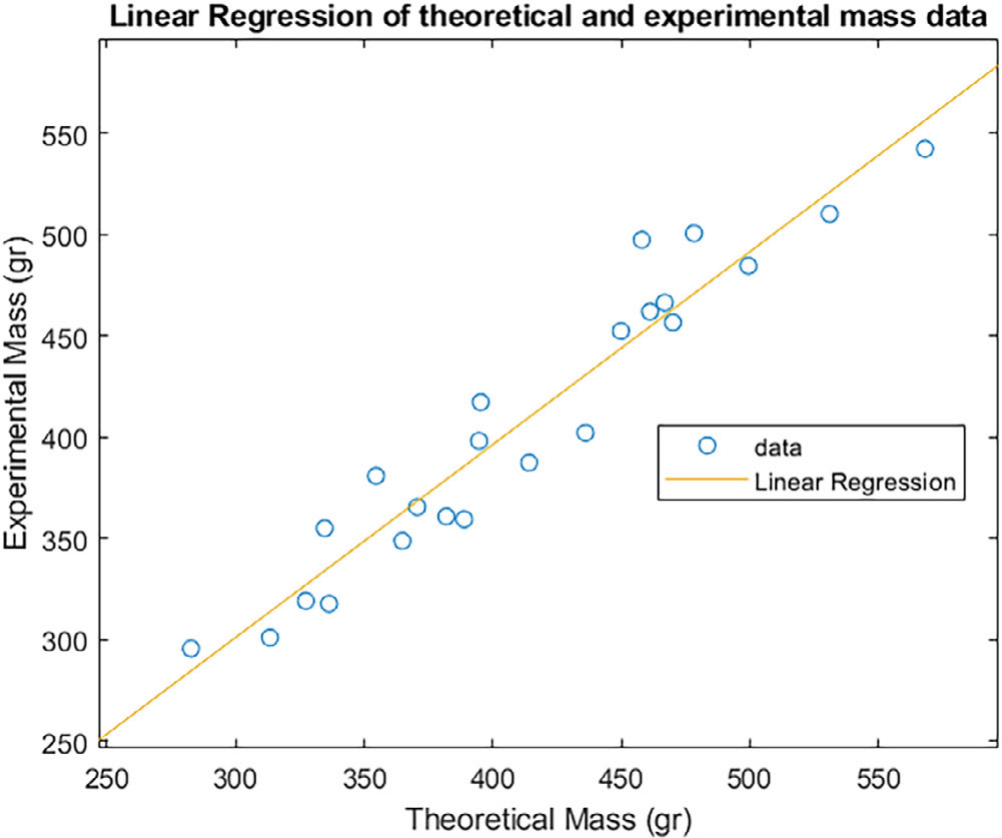
Regression analysis between the theoretical and experimental data for the total hand mass.

Table 1 shows the theoretical and experimental hand mass values calculated using (1), (2) and (3), respectively. To verify the correlation and agreement between the two different methods, a regression analysis alongside a Bland‐Altman plot was applied [12, 13, 14]. Figure 5 shows the linear regression plot between the theoretical and experimental methods with a fixed intercept at zero. In order to ensure that the theoretical mass aligns with the experimental mass, when the experimental mass is zero, and vice versa, the intercept of the linear regression was constrained to zero.

**TABLE 1:** Table of hand mass values determined from the theoretical calculations using cylindrical and parallelepiped approximations and the mean value of the experimental procedure alongside its standard deviation (SD).

Theoretical mass (g)	Mean experimental mass (SD) (g)
470.0488	456.5537 (7.8)
457.9147	497.2758 (59.7)
370.57367	365.5687 (12.3)
414.1126	387.4423 (17.18)
394.6050	398.1464 (13.8)
282.57598	295.7593 (24.97)
334.5856	355.0973 (35.97)
354.5880	380.9268 (17.27)
388.8874	359.5186 (16.35)
381.9015	360.9147 (13.25)
364.8193	348.8144 (9.57)
466.8331	466.327 (6.23)
395.3026	417.2277 (11.77)
436.0255	402.1023 (12.89)
478.2370	500.5336 (30.21)
336.3055	317.8656 (15.77)
461.1445	461.9057 (24.08)
531.0051	510.0742 (8.21)
499.4045	484.4774 (11.18)
313.3126	301.1113 (22.7)
327.2883	319.2618 (12.44)
568.0821	542.1866 (9.9)
449.8305	452.3651 (11.05)

The results from the regression analysis showed strong correlation between the two methods (Pearson's r = 0.9618, 95% confidence interval CI = 0.9105 − 0.9839, *p* < 0.00001). The corresponding regression equation is given by (4):

(4) $$y=b\left(95\%\;\mathrm{CI}\right)*x$$

with slope *b* = 0.9887 (0.9682, 1.0095). There is also significant corresponding goodness of fit ($R^{2}$ = 0.92).

For any two methods to be used interchangeably, strong correlation alone is not enough. The correlation coefficient measures the strength of the relationship between the two variables, but not their agreement [14]. ‘Perfect’ agreement is achieved if all the points in Figure 5 lie along the line of equality (Method A = Method B) [14]. To quantify the level of agreement between the two methods and ascertain any bias in the results obtained a Bland‐Altman analysis was performed. For a Bland‐Altman analysis the differences between the two methods under consideration should be normally distributed [12, 13, 14]. To check for normality, a Shapiro‐Wilk test was used. The *p*‐value from the Shapiro‐Wilk test was 0.2824 (> 0.05) suggesting that, at the 5% confidence level, the difference data follow a normal distribution. Since the differences between the two methods follow a normal distribution, then a Bland‐Altman plot can be used. This plot has on its x‐axis the mean of the two methods (Method A + Method B)/2 and on its y‐axis the difference between the two methods (Method A − Method B). The bias is quantified as the distance of the mean value of the differences $\bar{d}$, from the line of equality (Method A − Method B = 0) [12, 13, 14]. Let s, be the standard deviation of the differences and n be the sample size, then the 95% CI of the bias can be estimated from the standard error as shown in (5).

(5) $$\;{\bar{d}}_{C.I.}=\bar{d}\;\;\pm t_{n-1}\sqrt{s^{2}/n}$$

where $t_{n-1\;}$ is the point of the two‐tailed Student's t‐distribution with (*n −* 1) degrees of freedom (DOF) that can be found in tables for the t‐distribution at the confidence level *a =* 0.05 [12, 14]. Since the sample size for this experiment is 23 then the value from the two tailed Student's t‐distribution is taken with 22 DOF and has a value of $t_{22}=2.074$ [15]. The bias is then statistically significant if the line of equality is not within the range of the 95% CI of the bias [12].

To quantify the agreement between the two methods the limit of agreement (LOA) was calculated, where 95% of the differences are included. Equation (6) shows how the LOA was calculated [12, 13, 14]:

(6) $$\mathrm{LOA}=\bar{d}\;\;\pm 1.96*s$$

Similar to (5), it is possible to estimate the 95% CI of the LOA from (7) [12, 14]:

(7) $$\mathrm{LO}\;A_{C.I.}=\mathrm{LOA}\;\pm t_{n-1}\sqrt{3s^{2}/n}$$

**FIGURE 6:**
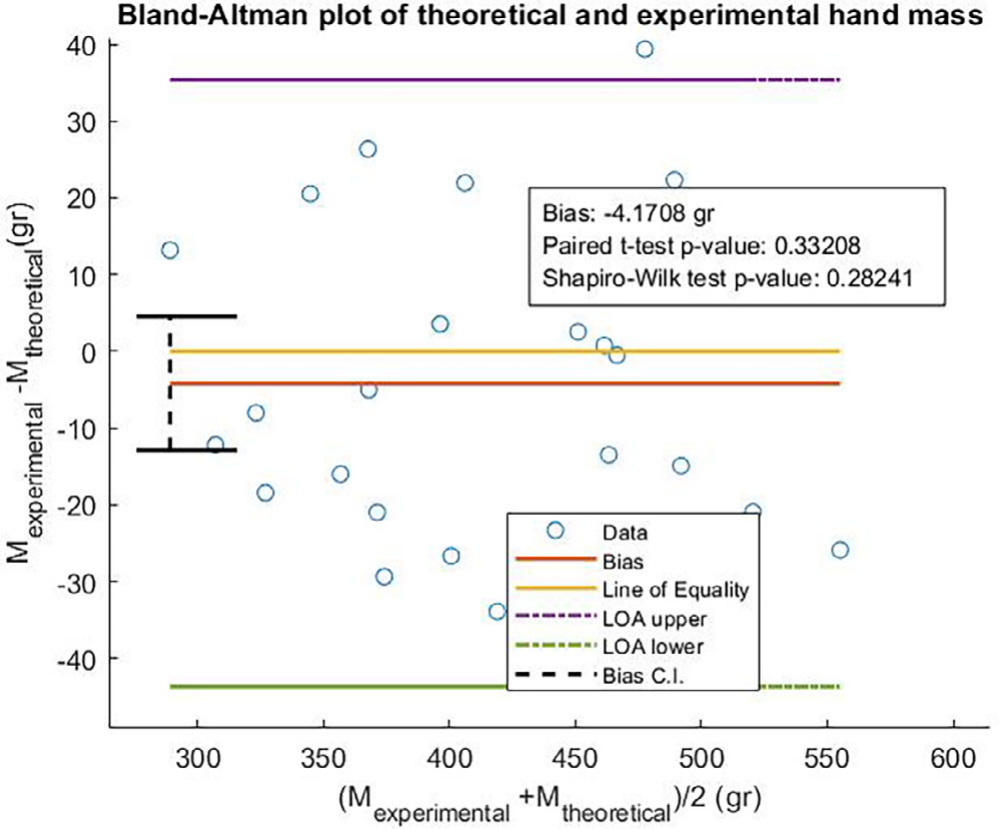
Bland‐Altman plot of the weight data from Table 1. The bias that corresponds to the mean of the differences is 4.2 g, *p*‐value = 0.33 and the limits of agreement are 44 and −35 g.

Figure 6 shows the Bland‐Altman plot for the hand weight data presented in Table 1.

The bias, which is estimated as the mean of the differences between the two methods is −4.17 g. The bias can be interpreted as follows: on average the theoretical method will measure 4.17 g more than the experimental method. The next step is to verify whether this bias is statistically significant. The estimated 95% CI for the bias, using (5), is (−4.5510, 12.8926) g. The line of equality is within the 95% CI of the bias and hence the bias is not considered as statistically significant [12]. This is evident from the *p*‐value for the bias, as shown in Figure 6. The *p*‐value arises from a paired t‐test that tests whether the mean of the differences is zero. Since the *p*‐value is 0.33, which is greater than the 0.05 confidence level, then the bias is not statistically significantly different from zero at the 5% confidence level, and thus both methods can be used interchangeably.

In terms of the agreement between the two methods, the following analysis was performed. The upper and lower LOAs (95% CI) are calculated from (6) and (7):

$$\mathrm{LO}A_{\mathrm{upper}}=44\;\left(28.593,\;58.806\right)\;g$$

$$\mathrm{LO}A_{\mathrm{low}}=-35\;\left(-50.465,\;-20.252\right)\;g$$

It is evident from these values that the agreement between the two methods is good. 95% of the differences between the two methods will be within 39.52g. Even though this value is perhaps slightly high, it corresponds to a maximum percentage error of 9.59%, assuming that the mass of a hand is estimated from the mean mass of the theoretical values. The LOA value is associated with possible errors in the estimate of the hand mass, either from the experimental procedure and/or the theoretical calculations, alongside the sample size. By increasing the sample size, more accurate estimates of the limits of agreement could be obtained. Despite this, the level of agreement is deemed acceptable for the estimation of total hand mass in‐vivo.

The determination of the finger segment length and radius based on the dimensions of the palm is now considered. The motivation for performing such measurements is to determine the length and radius of missing segments for partial hand amputees. Following the work of [7], linear regression analysis was performed between the finger segment length/radius and the palm dimensions alongside the hand length (HL), which is defined as the palm length plus the length of the middle finger. The set of scaling equations used for determining finger segment lengths from palm dimensions is shown below (8)–(13). The a,b,c parameters correspond to the regression coefficients.

(8) $$\;L_{i}=a*HL_{i}$$

(9) $$\;L_{i}=a*HB_{i}$$

(10) $$\;L_{i}=a*HW_{i}$$

(11) $$\;L_{i}=a*\left(PL_{i}+HB_{i}+HW_{i}\right)$$

(12) $$\;L_{i}=a*PL_{i}+b*HB_{i}+c*HW_{i}$$

(13) $$\;L_{i}=a*HL_{i}+b*HB_{i}+c*HW_{i}$$

The set of scaling equations used for determining finger segment radius from palm dimensions is shown below (14)–(19):

(14) $$\;R_{i}=a*HL_{i}$$

(15) $$\;R_{i}=a*HB_{i}$$

(16) $$\;R_{i}=a*HW_{i}$$

(17) $$\;R_{i}=a*\left(PL_{i}+HB_{i}+HW_{i}\right)$$

(18) $$\;R_{i}=a*PL_{i}+b*HB_{i}+c*HW_{i}$$

(19) $$\;R_{i}=a*HL_{i}+b*HB_{i}+c*HW_{i}$$

The primary difference between the scaling equations used in this paper and those presented in [7] is the inclusion of a broader range of palm dimensions, whereas the authors in [7] focused solely on the HL. To identify the equations that best fitted the data, a custom MATLAB script was used to calculate the coefficient of determination (*R^2^*) for each of the Equations (8) to (19) shown above across all finger segment dimensions. For each segment dimension, the scaling equation with the highest *R^2^* value was selected, consistent with the metric used in [7] and is presented in Tables 2 and 3 for the segment length and radius, respectively. All dimensions used in the analysis below are in millimetres.

**TABLE 2:** Regression coefficients for scaling the segment length, alongside the RMSE, $R^{2}$ and equation number.

Segment	a	b	c	Eq	$R^{2}$	RMSE (mm)
Thumb metacarpal	0.214	0	0	(11)	0.38	3.65
Thumb proximal	0.448	−0.53	−0.03	(13)	0.55	2.54
Thumb distal	0.158	0.02	−0.01	(13)	0.55	2.07
Index proximal	0.167	0.12	−0.1	(13)	0.17	3.58
Index middle	0.136	−0.01	0.105	(13)	0.34	1.91
Index distal	0.093	0.043	0.13	(13)	0.55	1.46
Middle proximal	−0.04	0.476	0.07	(12)	0.31	3.41
Middle middle	0.067	0.245	0.166	(12)	−0.1	2.47
Middle distal	0.045	0.175	0.232	(12)	0.38	1.63
Ring proximal	0.258	0.038	−0.46	(13)	0.32	4.14
Ring middle	0.165	0.045	−0.14	(13)	0.39	1.41
Ring distal	0.126	0.025	0.013	(13)	0.69	1.15
Little proximal	0.061	0.293	−0.26	(13)	0.46	2.27
Little middle	0.134	−0.01	−0.1	(13)	0.14	1.84
Little distal	0.125	0.02	−0.05	(13)	0.37	1.36

**TABLE 3:** Regression coefficients for scaling the segment radius, alongside the RMSE, $R^{2}$ and equation number.

Segment	a	b	c	Eq	$R^{2}$	RMSE (mm)
Thumb metacarpal	0.08	0.024	0.045	(19)	−0.1	1.64
Thumb proximal	0.03	0.04	0.114	(18)	0.61	0.5
Thumb distal	0.01	0.074	0.069	(19)	0.48	0.76
Index proximal	0.02	0.044	0.071	(19)	0.53	0.55
Index middle	0.03	0.046	0.005	(19)	0.34	0.66
Index distal	0.02	0.048	−0.01	(19)	0.53	0.53
Middle proximal	0.03	−0.01	0.116	(19)	0.45	0.56
Middle middle	0.04	0.005	0.048	(19)	0.48	0.55
Middle distal	0.024	0.034	0.027	(19)	0.53	0.39
Ring proximal	0.04	0.002	0.165	(18)	0.46	0.65
Ring middle	0.05	0.031	0.035	(18)	0.44	0.51
Ring distal	0.03	0.051	0.023	(18)	0.61	0.41
Little proximal	0.02	0.039	0.106	(18)	0.58	0.64
Little middle	0.02	0.024	0.061	(19)	0.33	0.57
Little distal	0.005	0.068	0	(19)	0.5	0.45

In the Appendix, tables are provided that show the scaling equations determined from the MATLAB script alongside the linear regression coefficients, the root mean square error (RMSE) and the $R^{2}$ for both the segment lengths and radii. The determined coefficients are different for each fit and for each equation.

## 4 Discussion

**FIGURE 7:**
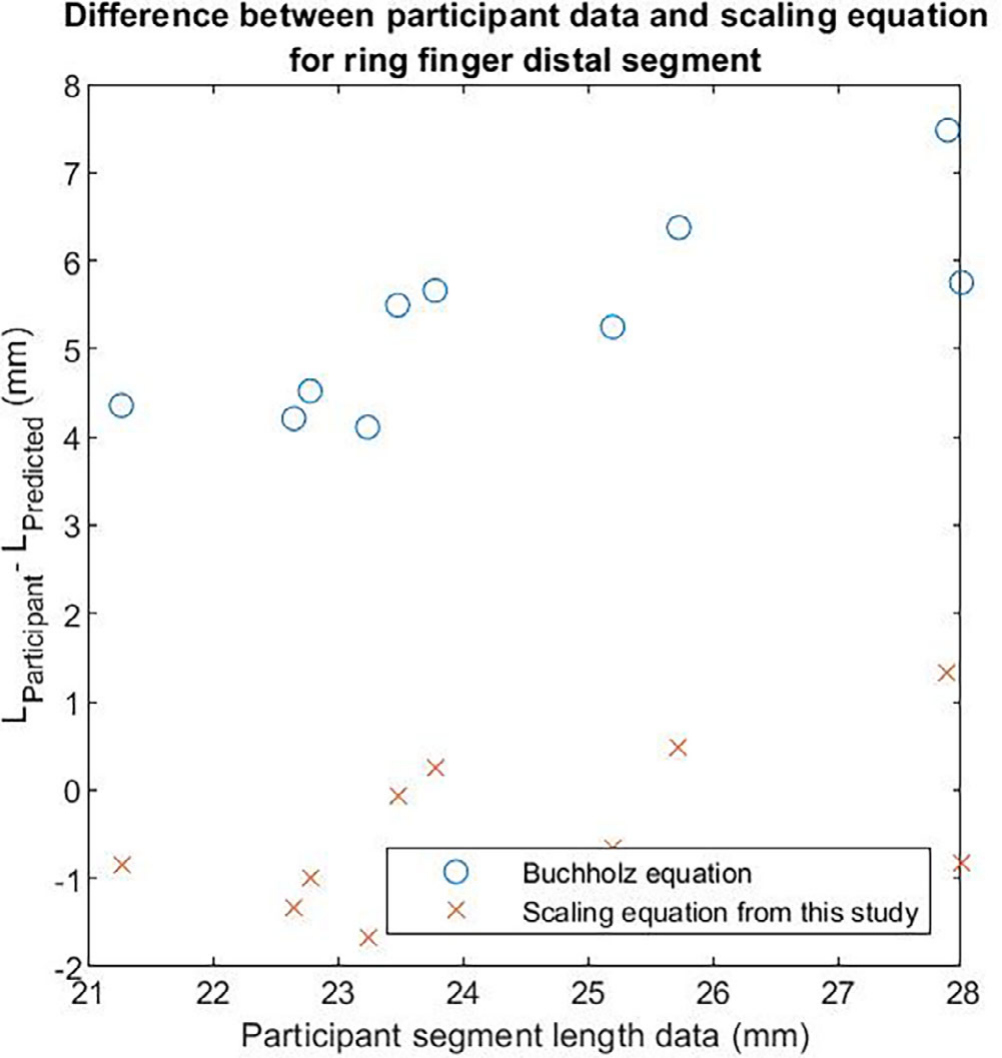
Plot of differences between participant and predicted values from Buchholz and this paper scaling functions for the ring finger distal segment length.

In this section a discussion on the results is presented, starting with the scaling functions found in Table 2 in the Appendix. These equations determine the dimensions of missing finger segments for partial hand amputees based on the residual dimensions of the palm. Considering the results presented in Table 2 and comparing these to the results provided in [7], it is evident that segment length is not dependent on just the hand length alone. In this study a set of different equations has been used to determine the optimal scaling functions, compared to [7], where only the hand length was used. These novel scaling functions have the potential to be used to determine the dimensions of missing segments for partial hand amputees. Recalling that hand length is the summation of the palm length and the entire length of the middle finger, the scaling functions provided in [7] cannot be used when someone has an amputation of their middle finger. Using the equations in [7] and the scaling equations in this study, we can compare the two methods against the participant segment measurements. To that end, 10 participants were recruited from the University of Warwick as part of the BSREC: 47.22/23 study. Participants in that study had their finger segment lengths and radii, alongside palm dimensions measured using the same principles described in the methods section. Then the palm dimensions of these 10 participants were used to determine each segment length from the two sets of equations and were compared against the measured ones from the experiment. In Figure 7 the differences between the participant data and the predicted values from Buchholz [7] and the scaling functions presented in this paper for the ring finger distal segment length can be seen.

As evident from Figure 7, the Buchholz scaling function constantly underestimates the ring finger distal segment length compared to the scaling Equation (13) presented in this paper, which slightly overestimates the same finger segment length. The highest difference in predictions is 7.5 mm for the participant segment length of 27.88 mm from Buchholz, compared to the prediction made from this study's scaling function, which is only 1.3 mm for the same length. To assess whether the scaling function (13) can be used as a predictor of the participant segment length, a one‐way ANOVA test was conducted. In Table 4 the pairwise comparison between actual length data and the ones predicted using the Buchholz functions from [7] and the ones determined in this paper is shown. To perform the One‐way ANOVA test, the data must follow a normal distribution. If that condition is met, then the one‐way ANOVA test is computed. If that condition is not met, then the Kruskal‐Wallis non‐parametric test is computed instead [16]. Results obtained from the Kruskal‐Wallis test are marked with an asterisk (*). When the *p*‐values shown in Table 4 are less than 5%, then there is a statistically significant difference between the means of the pairwise comparison. If the *p*‐values are greater than 5%, then the means of the pairwise comparison is not statistically different. Lastly, based on the pairwise comparisons, the proposed preferred scaling function is highlighted.

**TABLE 4:** Pairwise table comparison from the One‐way ANOVA test between the participant data and predicted data from Buchholz and this study scaling functions for segment lengths. The asterisk (*) indicates that the Kruskal‐Wallis non‐parametric test was used to obtain the p‐values.

Segment	Participant‐Buchholz *p*‐value	Participant‐this study *p*‐value	Buchholz‐this study *p*‐value	Proposed scaling function
Thumb metacarpal	0.195	0.783	0.053	Both
Thumb proximal	0.957	0.325	0.476	Both
Thumb distal	0.862	0.269	0.544	Both
Index proximal	0.107	0.013	<0.01	Buchholz
Index middle	0.923*	0.772*	0.535*	Both
Index distal	<0.01	0.69	<0.01	This study
Middle proximal	<0.01	0.109	<0.01	This study
Middle middle	—	—	—	Buchholz
Middle distal	<0.01	0.989	<0.01	This study
Ring proximal	<0.01	0.714	<0.01	This study
Ring middle	0.768	0.982	0.865	Both
Ring distal	<0.01	0.869	<0.01	This study
Little proximal	<0.01	0.362	<0.01	This study
Little middle	0.374	0.845	0.701	Both
Little distal	<0.01	0.517	<0.01	This study

For the middle finger middle segment, the pairwise comparison was not included since the $R^{2}$ value for the scaling function determined in this study was negative (as evident in Table 2), and hence the default choice for the determination of that finger segment length is the Buchholz scaling function.

**FIGURE 8:**
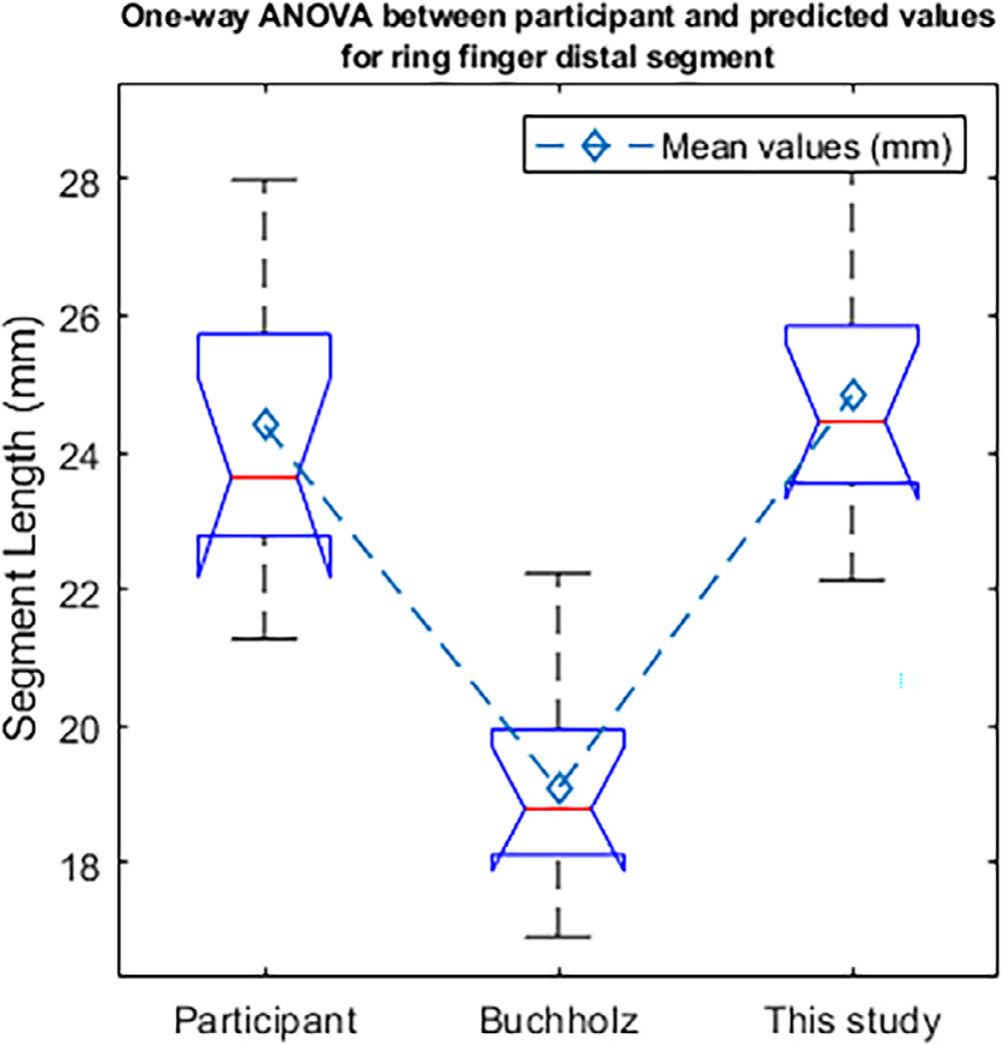
One‐way ANOVA test between participant and predicted values from Buchholz and this paper scaling function for the ring finger distal segment length.

In Figure 8 the one‐way ANOVA test can be seen for the participant data and predicted values for the ring finger distal segment length.

From Figure 8 it is evident that the mean of the Buchholz scaling function for the ring finger distal segment length is less than the mean of the participants' value and the mean of the values determined from the scaling function (13) in this paper. The pairwise comparison between the participant and data obtained from scaling function (13) shows that there is not a statistically significant difference between the means of the two approaches, as evident from the pairwise *p*‐value of 0.869, which is greater than the 5% confidence level. In contrast, the pairwise comparison between participant data and the Buchholz equation from [7] yielded a *p*‐value of less than 0.01, suggesting that the means of the two groups are statistically different. This suggests that scaling function (13) from this study can yield predictions that closely match the actual data for the ring finger distal segment.

From the results shown in Table 4, it is apparent that the scaling functions introduced in this study provide better or similar estimates of the lengths of the finger segments compared to those presented in [7], despite lower $R^{2}$ values. This is attributed to the reduced sample size considered in [7], where data from only six cadavers were used, compared to the 23 participants who took part in the present study for the determination of the scaling functions. Furthermore, in [7] the authors only investigated how the segment length changes with respect to hand length and did not consider any combinations of other palm dimensions. Similar outcomes to those for the middle finger scaling equations can be seen for the rest of the fingers in the hand, as shown in the plots provided in the supplementary material. Overall, the scaling equations introduced in this study provide better estimates of segment lengths compared to the ones found in [7] for partial hand amputees and across the whole of the hand.

The in‐vivo hand mass calculation yields a strong correlation between the theoretical and experimental methods, as shown in Figure 5. This is further supported by the Bland‐Altman analysis shown in Figure 6, which reveals that the mean difference between the two methods is not statistically significant, suggesting that they can be used interchangeably. This outcome implies that the geometric approximations applied in the methods section are statistically valid and can be used to infer the mass of finger segments and the palm in a human hand. Despite the strong statistical correlation, there are limitations in the methods and approaches presented. For example, hand density data often rely on decades‐old studies, and specific density values for the fingers and palm are rarely available in the literature. In this study, we assumed a uniform density for the palm and fingers, which may not reflect actual variations. Future studies are thus required to determine specific densities for the fingers and palm, and to assess potential differences across individuals and genders. Additionally, the water displacement method has limitations, primarily due to the assumed hand density. If densities of the fingers and palm differ, determining total hand mass accurately becomes challenging with this method. However, this approach remains valuable for validating geometric hand approximations by determining hand volume rather than mass. The geometric approximations presented in this study may also serve as a useful guide for designing prosthetic finger segments for partial hand amputees, as they enable the determination of mass for missing segments, with dimensions derived from the provided scaling equations. It is worth noting that these scaling equations are mathematical constructs, and some deviations from actual values are inevitable. Nevertheless, these equations offer an updated set of scaling guidelines for the prosthetics literature on partial hand amputees. Despite these limitations, the geometric approximations presented here have been shown to be statistically valid, offering potential applications in both prosthetic finger design and musculoskeletal modelling of the hand. The latter is further demonstrated in [17], where a mathematical model describing the fingers as a serial linkage of three cylindrical pendula was developed. The cylindrical approximation of the human fingers used in that model is valuable for calculating the moments of inertia of the finger segments, which are subsequently used as known parameters in the parameter estimation procedure.

## 5 Conclusion

The scaling equations determined from the anthropometric measurements, as shown in Tables 2 and 3, can serve as support for prosthetic finger design and offer the potential to tailor prostheses to the anthropometry of the individual. Furthermore, from the results presented in this paper, it can be concluded that both approximations presented for determining the mass of the hand can be considered acceptable, since there is a strong correlation and good agreement between recorded experimental outcomes and the theoretical methods applied. To determine the mass of missing finger segments for prosthetic design, the proposed approach first estimates the dimensions of the missing segments from residual palm measurements and subsequently infers segment mass using a cylindrical geometric approximation. The presented scaling equations therefore enable estimation of finger segment length, radius, and mass directly from palm measurements, supporting reconstruction or prosthetic design without requiring prior knowledge of intact hand dimensions in partial hand amputees.

## Author Contributions

**Panagiotis Tsakonas**: conceptualisation, data curation, formal analysis, investigation, methodology, software, validation, visualisation, writing – original draft, writing – review and editing. **Neil D. Evans**: conceptualisation, funding acquisition, methodology, project administration, supervision, writing – original draft, writing – review and editing. **Joseph Hardwicke**: conceptualisation, funding acquisition, project administration, supervision, writing – original draft, writing – review and editing. **Shervanthi Homer‐Vanniasinkam**: conceptualisation, funding acquisition, methodology, supervision, writing – original draft, writing – review and editing. **Michael J. Chappell**: conceptualisation, funding acquisition, investigation, methodology, project administration, supervision, writing – original draft, writing – review and editing.

## Funding

This work was supported by the EPSRC Standard Research Studentship (DTP) under grant EP/T51794X/1, EP/V011375/1, EP/V010808/1, EP/V01062X/1.

## Conflicts of Interest

The authors declare no conflicts of interest.

## Data Availability

The data for segment lengths and radii are not available due to ethical restrictions. Additional figures can be found in the supplementary material.
