# Correction to ‘In‐Vivo Hand Mass Determination and an Anthropometric Investigation on Segment Length and Radius for Prosthetic Segment Design’

**DOI:** 10.1049/htl2.70086

**Published:** 2026-05-08

P. Tsakonas, N. D. Evans, J. Hardwicke, S. Homer‐Vanniasinkam, and M. J. Chappell, “In‐Vivo Hand Mass Determination and an Anthropometric Investigation on Segment Length and Radius for Prosthetic Segment Design,” *Healthcare Technology Letters* 13, no. 1 (2026): e70078, https://doi.org/10.1049/htl2.70078

Figure 1 was depicting an anatomically incorrect human hand skeleton, and the arrows indicating the landmarks for measuring segment diameters were not shown in the published version. The revised Figure 1 can be seen below, which has been adapted from [1].

**FIGURE 1:**
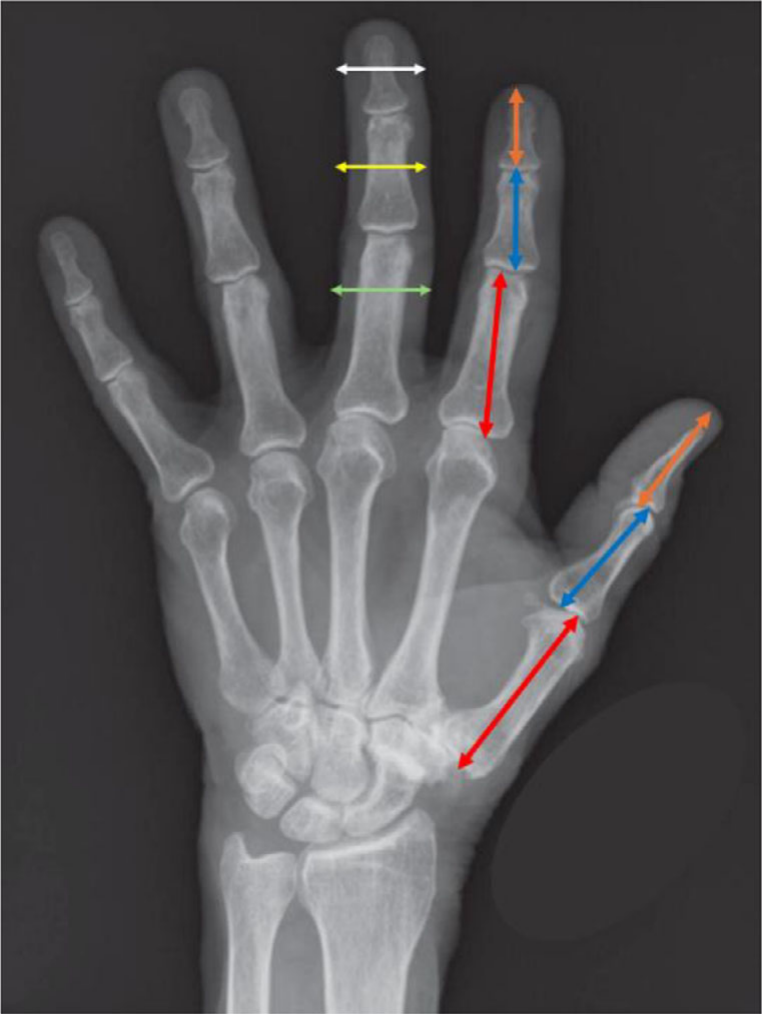
Landmarks for measurements of the finger segments. The orange arrow corresponds to the landmarks for the measurement of the distal segment. The blue arrow corresponds to the landmarks used for the middle segment, and the red arrow corresponds to the landmarks used for the measurement of the proximal segment. The white arrow shows the landmarks for the measurement of the diameter of the distal segment. The yellow arrow shows the landmarks for the measurement of the diameter of the middle segment, and the green arrow shows the landmarks for the measurement of the diameter of the proximal segment. *Source*: Wolfe et al. [1].

[1] S. W., Wolfe, W. C., Pederson, S. H., Kozin, M. S. Cohen, *Green's Operative Hand Surgery*, 7th ed. (Elsevier Health Sciences, 2016).

We apologize for this error.

